# Diversity and Antimicrobial Activity of Endophytic Fungi Isolated from *Chloranthus japonicus* Sieb in Qinling Mountains, China

**DOI:** 10.3390/ijms21175958

**Published:** 2020-08-19

**Authors:** Chao An, Saijian Ma, Xinwei Shi, Wenjiao Xue, Chen Liu, Hao Ding

**Affiliations:** 1 Shaanxi Institute of Microbiology, Xi’an 710043, China; anchor0216@sina.com (C.A.); masaijian@163.com (S.M.); lcmomo@126.com (C.L.); dhwj2010@126.com (H.D.); 2 Engineering Center of QinLing Mountains Natural Products, Shaanxi Academy of Sciences, Xi’an 710043, China; sxw@ms.xab.ac.cn; 3 Xi’an Botanical Garden of Shaanxi Province (Institute of Botany of Shaanxi Province), Xi’an 710061, China

**Keywords:** endophytic fungi, diversity, antimicrobial activity, *Chloranthus japonicus*, Qinling Mountains

## Abstract

The plant *Chloranthus japonicus* Sieb is known for its anticancer properties and mainly distributed in China, Japan, and Korea. In this study, we firstly investigated the diversity and antimicrobial activity of the culturable endophytic fungi from *C. japonicus*. A total of 332 fungal colonies were successfully isolated from 555 tissue segments of the medicinal plant *C. japonicus* collected from Qinling Mountains, China. One hundred and thirty representative morphotype strains were identified according to ITS rDNA sequence analyses and were grouped into three phyla (Ascomycota, Basidiomycota, Mucoromycota), five classes (Dothideomycetes, Sordariomycetes, Eurotiomycetes, Agaricomycetes, Mucoromycetes), and at least 30 genera. *Colletotrichum* (RA, 60.54%) was the most abundant genus, followed by *Aspergillus* (RA, 11.75%) and *Diaporthe* (RA, 9.34%). The Species Richness Index (*S*, 56) and the Shannon-Wiener Index (*H*′, 2.7076) indicated that *C. japonicus* harbored abundant fungal resources. Thirteen out of 130 endophytic fungal ethyl acetate extracts exhibited inhibitory activities against at least one pathogenic bacterium or fungus. Among of these, F8158, which was identified as *Trichoderma* cf. *harzianum*, exhibited good antagonistic capacities (the percent inhibition of mycelial growth ranged from 47.72~88.18) for different pathogens and has a potential application in biological control. In addition, it is noteworthy that the strain F8157 (*Thanatephorus cucumeris*, an opportunistic pathogen) showed antibacterial and antifungal activity, which is reported firstly in this study, and should be investigated further. Taken together, these results indicated that the endophytic fungi from *C. japonicus* may be of potential interest in screening bio-control agents and discovering of new bioactive compounds.

## 1. Introduction

The Qinling Mountains (32°30′−34°45′ N, 104°30′−112°45′ E), which are mainly located in the south of Shaanxi province in central China, are the most important natural climatic boundary between the subtropical and warm temperate zones of China, and support an astonishingly high biodiversity [1,2,3,4]. In particular, the Qinling Mountains are extremely rich in medicinal plants, including *Sinopodophyllum hexandrum* (Royle) Ying, *Rohdea chinensis* (Baker) N.Tanaka, *Bergenia scopulosa* TP Wang, *Aconitum taipeicum* Hand-Mazz, etc. *Chloranthus japonicus* Sieb. (Chloranthaceae, “yin-xian-cao” in Chinese), which is a perennial herb mainly distributed in eastern Asia such as the mainland of China, Korea, and Japan [5], is also a typical medicine resource in Qingling Mountains. Its whole plants have long been a Chinese folk medicine to treat traumatic injuries, rheumatic arthralgia, fractures, pulmonary tuberculosis, and neurasthenia in China [6]. It was reported that the sesquiterpenoids were its mainly active compounds in this plant [5,6,7].

Endophytic fungi, which inhabit the interiors various plant tissues without causing disease or injury to the host, have a very complex relationship with their host plants [8]. The endophytic fungi not only produce hormones that promote plant growth and help the host resist abiotic stress but also produce bioactive secondary metabolites, including those originated from the host plants [9,10]. For instance, all the endophytic fungi taxa isolated from the *Glycyrrhiza glabra* have been reported to produce the plant growth promoting hormone indole acetic acid (IAA) [11]. Taxol, a widely employed anticancer drug, was originally isolated from the bark of *Taxus brevifolia*, and have also been produced by a series of endophytic fungi from different plants [12,13]. Tan et al. isolated two endophytic fungi producing the podophyllotoxin, a main active compound from the host plant *Dysosma versipellis* [14].

Due to the increase in antibiotic resistance among pathogens, discovering novel antimicrobials is urgent [15]. Endophytic fungi represent abundant species resources and secondary metabolites diversity [16]. Nevertheless, the resources of endophytic fungi remain underexplored. In this study, our main purpose is to investigate the diversity of these endophytic fungi from *C. japonicus* and to obtain endophytic fungi resources with antibacterial activities, so as to provide a basis for further screening for biocontrol agents or mining natural products with new structures. To the best of our knowledge, this is the first report on the diversity and antimicrobial activity of endophytic fungi isolated from *C. japonicus*.

## 2. Results

### 2.1. Isolation, Sequencing, Identification, and Diversity Analyses of the Endophytic Fungi from C. japonicus

In this study, a total of 332 fungal colonies were successfully isolated from 555 tissue segments of *C. japonicus* with a potato dextrose agar (PDA) medium. The 332 isolates initially were assigned to 130 representative morphotypes according to their culture characteristics on PDA. ITS rDNA sequences subsequently were generated for a representative of each morphotype (Supplementary Information Files (1)). Based on the sequence similarity threshold (SSA, 97%~100%) 124 isolates were categorized at the genus level while the other six isolates remained unidentified. The phylogenetic trees were constructed by using the maximum likelihood method (Supplementary Information Files (2)). Some species were still not well categorized. For example, although the isolates F8210, F8235, F8210, F8176, F8170, F8135, F8110, F8115, and *Colletotrichum* sp. SL10 (KP689235) were in the same branch, the support rating was only 60%. These isolates may be different taxa though we classified them into one taxon (*Colletotrichum* sp. SL10) in this study. In total, these isolates were grouped into at least 56 taxa (Table 1) based on the results of sequence BLAST and phylogenetic trees analysis. The isolate F4146 was only assigned to the phylum Ascomycota, and the isolates F8184, F8209, F8233, F8241, and F8245 were only categorized as the class Sordariomycetes. Ultimately, the Species Richness Index (S) and Shannon-Wiener Index (*H*′), which are two important parameters for diversity analysis, were at least 56 and 2.7076 for *C. japonicus*, respectively. As presented in Table 2, these isolates have been further grouped into three phyla (Ascomycota, Basidiomycota, Mucoromycota), five classes (Dothideomycetes, Sordariomycetes, Eurotiomycetes, Agaricomycetes, Mucoromycetes), 12 orders, 21 families, and at least 30 fungal genera.

### 2.2. The Relative Abundance (RA) Analyses of Endophytic fungi from C. japonicus

**Figure 1:**
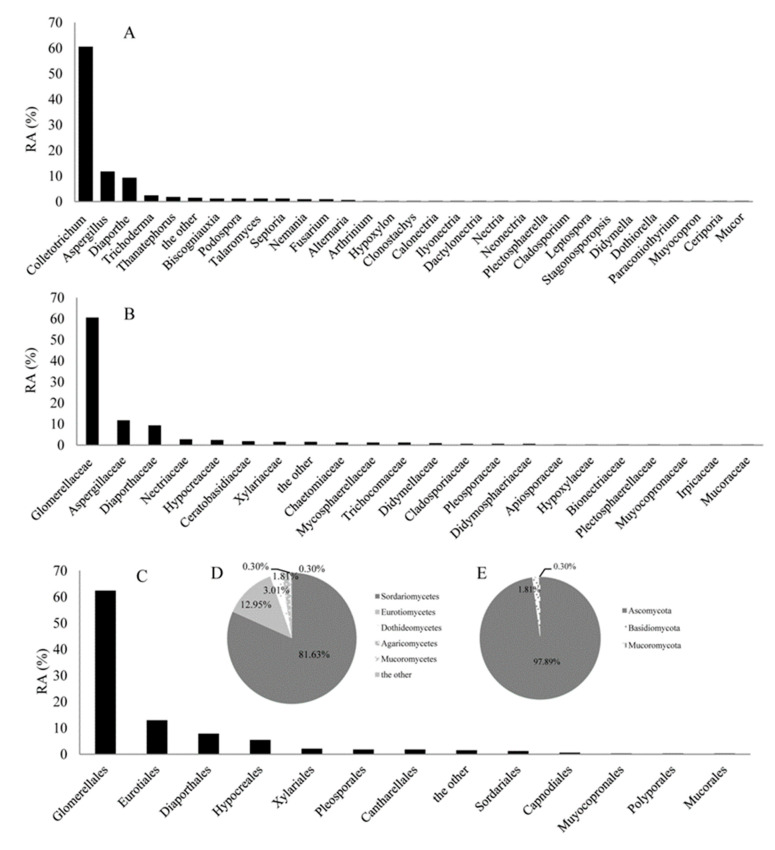
The relative abundance (RA, %) of endophytic fungi at the level of genus (**A**). Family (**B**), order (**C**), class (**D**), and phylum (**E**).

As shown in Table 1, *Colletotrichum truncatum*, *Colletotrichum acutatum,* and *Colletotrichum* sp. were dominant species and their IF were 32.83%, 16.57%, and 9.34%, respectively. The RA of these isolates at genus, family, order, class, and phylum levels were shown in Figure 1A–E, respectively. At the genus level, *Colletotrichum* (RA, 60.54%) was most abundant, followed by the *Aspergillus* (RA, 11.75%) and *Diaporthe* (RA, 9.34%). *Aspergillus* included at least eight species (*Aspergillus flavipes*, *Aspergillus terreus*, *Aspergillus pseudoglaucus*, *Aspergillus niger*, *Aspergillus oryzae*, *Aspergillus* sp., *Aspergillus tubingensis*, *Aspergillus flavus*); *Diaporthe* included at least seven species (*Diaporthe eres*, *Diaporthe longicolla*, *Diaporthe nobilis*, *Diaporthe gulyae*, *Diaporthe longicolla*, *Diaporthe vaccinii*, *Diaporthe citrichinensis*); *Colletotrichum* included at least seven species (*Colletotrichum boninense*, *Colletotrichum* sp., *Colletotrichum acutatum*, *Colletotrichum truncatum*, *Colletotrichum fructicola*, *Colletotrichum gloeosporioides*, *Colletotrichum higginsianum*). At the family level, Glomerellaceae (RA, 60.54%), Aspergillaceae (RA, 11.75%), and Diaporthaceae (RA, 9.34%) were the three most abundant groups in this study. At the phylum level, the majority of fungi isolated from *C. japonicus* were identified as Ascomycota (RA, 97.89%), which represented three classes (Sordariomycetes (RA, 81.63%), Eurotiomycetes (RA, 12.95%), and Dothideomycetes (RA, 3.01%)). In addition, one class (Agaricomycetes, RA, 1.81%) belonged to Basidiomycota (RA, 1.81%), and one class (Mucoromycetes, RA, 0.31%) belonged to Mucoromycota (RA, 0.31%).

### 2.3. Antimicrobial Activity Screening of the Ethyl Acetate Extracts from Endophytic Fungal Culture Filtrates

As shown in Table 3, 13 out of 130 endophytic fungal ethyl acetate extracts showed inhibitory activity against at least one pathogenic bacterium or fungus (Supplementary Information Files (3)). The other 117 extracts did not show inhibitory activities. These 13 strains belonged to genera of *Diaporthe*, *Trichoderma*, *Aspergillus*, *Leptospora*, *Thanatephorus*, *Colletotrichum*, *Septoria,* respectively (Table 2). Among these strains, F8158 (*Trichoderma* cf. *harzianum* (GenBank Accession number: MN429210), commonly used as biocontrol agents in the agricultural production process), also exhibited a broad spectrum antimicrobial activity and has potential application in the biological control. Further experiments showed that the strain F8158 exhibited a plant promoting effect by producing siderophore (data not shown). In addition, it is noteworthy that the strain F8157 (*Thanatephorus cucumeris*, an opportunistic pathogen), showed antibacterial and antifungal activity, which is reported firstly in this study, and has the potential research value.

### 2.4. Identification of the Strain F8158 and Analysis of Its Antagonistic Effect for Different Pathogens

**Figure 2:**
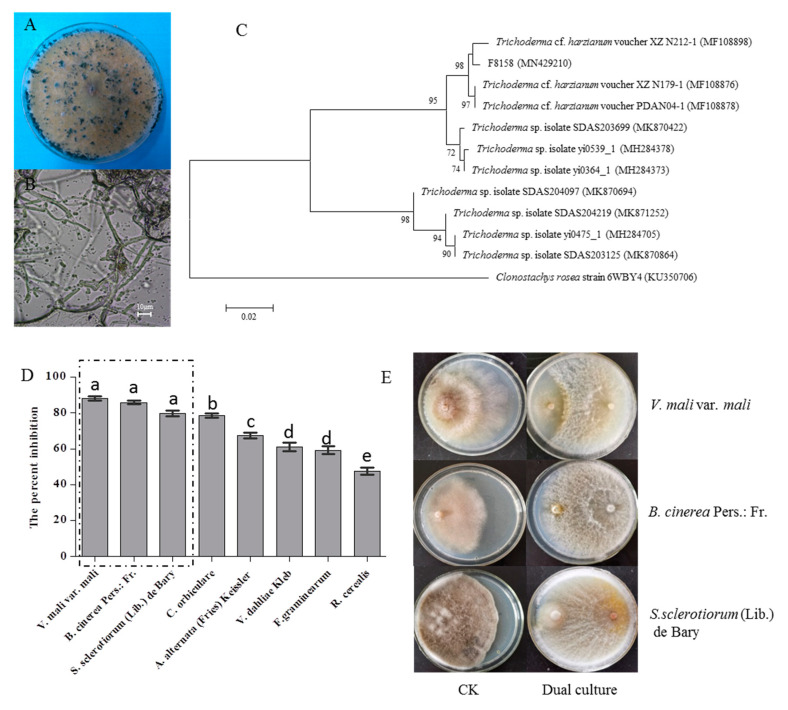
The characteristics of the strain F8158. The morphology of the colony (**A**) and microscopic morphology (**B**) on a potato dextrose agar (PDA) medium; phylogenetic identification based on ITS1 and ITS4 using the maximum-likelihood methods (**C**); the percent inhibition against different pathogens (**D**) and the good antagonistic activity against three pathogens (**E**) in the dual culture assays.

The colonial and microscopic morphology of the strain F8158 on PDA were shown in Figure 2A,B and were similar to those of *Trichoderma* spp. [17,18]. The isolated fungal culture F8158 was observed as fast growing with a scanty mycelial growth on PDA. During the growth process, mycelia of the strain F8158 spread out in all directions and the colony color turned from white to green. The fungal hyphae of F8158 were hyaline and smooth-walled and the conidiophores are highly branched. A subsequent sequence analysis showed that the isolate F8158 shared 99.66% identity with *Trichoderma* cf. *harzianum* voucher PDAN04-1 (MF108878) in the NCBI database (Figure 2C). The strain F8158 exhibited good antagonistic capacities (the percent inhibition of mycelial growth ranged from 47.72–88.18) against different pathogens (Figure 2D). In particular, the average growth percent inhibition of F8158 against *V. mali* var. *mali* was the highest and reached 88.17 ± 1.51, followed by *B. cinerea* Pers.: Fr. with a percent inhibition of 85.97 ± 1.27 and the percent inhibition of *S. sclerotiorum* (Lib.) de Bary was 79.87 ± 1.84 (Figure 2E).

## 3. Discussion

Endophytic fungi have a high level of taxonomic diversity [19]. These fungi exist in nearly every tissue type studied and are promising as biological control agents against phytopathogens and bioactive substances [10]. Despite these characteristics, endophytic fungi remain poorly studied. The Qinling Mountains are rich in medicinal plant resources, many of which have been used as traditional Chinese medicines by the local people. In recent years, increasing attention has focused on biodiversity and pharmacological properties of medicinal plants of the Qinling Mountains; however, as far as we know, few studies have attempted to evaluate the diversity of endophytes associated with these valuable plants [20]. In this study, we investigated the diversity of the culturable endophytic fungi from *C. japonicus* (one of the most popular medicinal plants in Qinling Mountains) and obtained abundant endophytic fungal isolates, which was conducive for screening the active strains and laying a foundation for their application.

At present, the discovery of new compounds was urgent but very difficult from common environments (e.g., soils) [21,22,23]. Researchers have focused on the selection of antimicrobial substances from microorganisms in other ecosystems, particularly those in special habitats, such as marine microorganisms and endophytes [24]. In our research, ethyl acetate extracts of 13 endophytic fungi (10% of total screened strains) showed inhibitory activity against pathogenic microorganisms. Most of the 13 isolates represent fungal genera, including *Aspergillus*, *Diaporthe*, and *Trichoderma*, previously reported to produce antimicrobial compounds [25,26,27]. However, new bioactive compounds are constantly being discovered from plant endophytes. For instance, a new cadinene-sesquiterpenes and seven of its analogues were isolated from an endophytic fungus, *Aspergillus flavus*, which was isolated from a toxic medicinal plant, *Tylophora ovate* [28]. Guo et al. isolated a new antibacterial secondary metabolite (Diaporone A) from the plant endophytic fungus *Diaporthe* sp. [29]. Seven previously unreported cyclonerane derivatives were isolated from the culture of *Trichoderma asperellum* A-YMD-9-2, an endophytic fungus obtained from the marine red alga *Gracilaria verrucosa* [30]. In addition, it is noteworthy that the strain F8157 (*Thanatephorus cucumeris*, an opportunistic pathogen) showed antibacterial and antifungal activity, which is reported firstly in this study, and has the potential research value.

To the best of our knowledge, this is the first report on the diversity and antimicrobial activity of endophytic fungi isolated from *C. japonicus*. Thus, the fungi with antimicrobial activity obtained in this study still have the potential to produce new structures natural products. In addition, F8158 exhibited higher antagonistic activity against *V. mali* var. *mali*, *B. cinerea* Pers.: Fr., and *S. sclerotiorum* (Lib.) de Bary compared with previous studies and have a potential application in biological control [31,32,33].

## 4. Materials and Methods

### 4.1. Source of Plant Samples

In April 2018, a total of thirty wild plants of *C. japonicus* were collected from Yingpan town, Shaanxi province of China (33°49′30′′ N, 109°6′15′′ E, elevation, 1340 m). The land we accessed was publicly owned and undeveloped. These plants were carefully dug up, placed in a sterile sampling bag, labeled, immediately transported to the laboratory, and then placed into a refrigerator (4 °C), as described previously [34]. All of the samples were used to isolate endophytic fungi within 48 h after collection.

### 4.2. Fungal Isolation and Cultivation

The plant tissues were processed with the method described by Qin et al. [35]. In brief, the samples were thoroughly washed in running water for 30 min, followed by an ultrasonic cleaning (200 W, 10 min), and then air-dried for 2 h at room temperature. After drying, the plant samples were surface-sterilized with the protocol described by Tan et al. with minor modifications [36]. Air-dried plant samples were surface-sterilized using sequential washes in 70% ethanol for 1 min, 2.5% NaClO_2_ for 2 min, and 70% ethanol for 1 min. Following sterilization, leaves were rinsed three times in sterile distilled water. All the samples were then excised into 555 segments of 1–2 mm length. Segments were placed on a PDA medium (house-made, containing (g/L): Agar power 16, potato 200 (peel potatoes, cut into 1 cm pieces, boiled for 20 min, filtered to obtain filtrate), and dextrose 20, pH 6.0) using 90 mm petri plates. The medium was supplemented with amikacin sulfate (100 U/mL) to prevent the growth of bacteria. Seven tissue segments were placed on each petri dish (90 mm), which were then sealed with parafilm and incubated at 28 °C for one week. Emergent fungal colonies were isolated and purified in a PDA medium for further identification and bioactive assays (see below). Pure isolates growing on the PDA medium were photographed and the agar piece plugs with pure isolates were stored at −80 °C in a 20% glycerol solution in the engineering center of QinLing Mountains natural products, Shaanxi provincial institute of microbiology.

### 4.3. Molecular Identification and Phylogenetic Analyses

To obtain fungal mycelia, each pure isolate was cultivated on plates containing the PDA medium at 28 °C for seven days. Mycelia were removed from media using sterile pipette tips and then ground in liquid nitrogen for DNA extraction using the TaKaRa MiniBEST Bacteria Genomic DNA Extraction Kit (Dalian, China). Genomic DNA was then used as the template for PCR amplification of the nuclear ribosomal DNA internal transcribed spacer (ITS) using the universal primers ITS1 (5′-TCCGTAGGTGAACCTGCGG-3′) and ITS4 (5′-TCCTCCGCTTATTGATATGC-3′) according to the description by White et al. with minor modifications [37]. The final reaction volume was 50 μL, containing 5.0 μL of 10×Taq buffers, 4.0 μL of 200 mmol/L dNTPs, 2.0 μL of each primer at 10 μM, 0.5 μL of Ex Taq enzyme (TaKaRa, Dalian), and 5.0 μL of genomic DNA. PCR amplification was performed using TProfessional Standard 96 Gradient (Biometra, Jena, Germany) using the following cycling parameters: 1 min 95 °C; followed by 35 cycles of 15 s at 95 °C, 30 s at 55 °C, and 1 min at 72 °C; and a final 10 m extension at 72 °C. Five μL of each PCR product was analyzed electrophoretically in 1% (*w/v*) agarose gels stained with GelRed (Shanghai Generay Biotech Co., Ltd., China). The PCR products were subsequently purified and sequenced by BGI Biotechnology (Shenzheng, China). The raw obtained sequences were aligned using MEGA 5.05 (Arizona State University, Tempe, AZ, USA), edited manually, and then BLAST (Basic Local Alignment Search Tool) (NCBI, Rockville Pike, Bethesda, MD, USA) was used to search for the best match in the National Center for Biotechnology Information (NCBI) GenBank database (http://www.ncbi.nlm.nih.gov/) to identify endophytic fungi. Sequences with similarity over 97% belonged to the same genus. The sequences obtained in this study were submitted to the GenBank database with accession numbers from MK367469 to MK367568. The evolutionary history was inferred as described by Wei et al. and Felsenstein [38,39]. All sequences were aligned by MEGA 5.05 using the alignment prepared with Clustal W (Arizona State University, Tempe, AZ, USA) and all positions containing gaps and missing date were deleted. Finally, the maximum likelihood phylogenetic trees were constructed for each of the families using MEGA software 5.05 [40].

### 4.4. Crude Extract Preparation of Fungal Fermentation Broth

Each isolate was cultured on the PDA for seven days, after which the plugs of each fungus were used to inoculate liquid cultures containing a 250 mL Erlenmeyer flask containing a 50 mL potato dextrose broth (PDB) culture medium (house-made) containing (g/L): 200 potato (peel potatoes, cut into 1 cm pieces, boiled for 20 min, filtered to obtain filtrate), 20 dextrose, pH 6.0. All isolates were incubated on a rotary shaker at 28 °C and 230 rpm for 14 days. The fermentation broth was collected by centrifugation at 8000 rpm for 8 min. Fifty mL of fermentation filtrate was extracted with 50 mL ethyl acetate (three extractions total) and the organic phase was concentrated using a rotary evaporator on 50 °C water bath to remove organic solvent as described by Xing et al. with minor modifications [41]. The crude extracts were diluted with pure methanol to 10 mg/mL and sterilized by filtration using an organic filter (0.22 μm, Shanyu Co., Ltd., Shanyu, China).

### 4.5. Antimicrobial Activity

Using the agar diffusion method described by Wang et al. [42], we screened the ethyl acetate crude extracts from fermentation filtrates of 130 fungal strains for antimicrobial activities against seven pathogens, including *Bacillus cereus*, *Escherichia coli*, *Bacillus subtilis*, *Staphylococcus aureus*, *Pseudomonas aeruginosa*, *Xanthomonas oryzae* pv. *oryzae,* and *Candida albicans*. The detailed operation procedure were as follows: 10 mL culture of the *C. albicans* grown two days in a Sabouraud liquid medium at 28 °C was added to the 200 mL of the Sabouraud agar medium, while 10 mL cultures of pathogenic bacteria grown 12 h in the Luria-Bertani (LB) liquid medium (containing (g/L): 5 Yeast extracts, 10 peptone, 10 NaCl, pH 7.0) at 28 °C was added to the 100 mL of the LB agar medium, mixed gently, and then poured slowly on the petri dish (90 mm) used as the test plate. Six mm sterilized straws were used for perforating the plate, and the agar blocks were removed with a sterilized toothpick. The 100 μL fermentation filtrate EtOAc extracts (10 mg/mL) were added to the hole of the test plate. The plates were incubated at 37 °C for 24 h for the pathogenic bacteria or at 28 °C for 48 h for *C. albicans*. Ampicillin sodium (1 mg/mL) and Actidione (1 mg/mL) were prepared using deionized water and used as a positive antimicrobial control and pure methanol was used as a negative control. Antimicrobial activities were evaluated by measuring the diameter of the inhibition zones. All experiments were replicated three times.

### 4.6. Dual Culture Assay to Detect the Antagonistic Potential of Endophytic Fungi against Different Pathogenic Fungi

The dual culture technique was used to conduct the antagonistic test. The endophytic fungi and pathogen species to be tested were cultured separately on the PDA for seven days. After seven days, 5 mm mycelial plugs (taken from the edge of fungal colonies) of each species were transferred to PDA plates using a borer. The mycelial plugs of endophytic fungi and pathogens were placed on opposite sides to each other on a PDA surface. PDA plates inoculated with the pathogens were included as negative controls. The antagonistic tests were conducted in duplicate. The controls consisted of pure *Botrytis cinerea* Pers.: Fr., *Rhizoctonia cerealis*, *Valsa mali* var. *mali*, *Colletotrichum orbiculare*, *Sclerotinia sclerotiorum* (Lib.) de Bary, *Fusarium graminearum*, *Verticillium dahliae* Kleb, and *Alternaria alternata* (Fries) Keissler cultures. All culture plates were incubated at 28 °C and observations were made after five days of incubations. The percent inhibition of mycelia growth over the control was calculated by the following equation:

I = (C − T)/C × 100(1)

where I is the percent inhibition of mycelial growth, C is the radius of pathogens mycelium with the growth in control, and T is the radius of pathogens mycelia in the treatment.

### 4.7. Diversity Analyses of the Endophytic Fungi

The isolation frequency (IF) represented the frequency of the occurrence of certain endophytic fungi in total isolates based on the number of isolates (N). The relative abundance (RA) was calculated based on the number of all isolates number (N). The diversity of fungal species from *C. japonicus* was evaluated using the Species Richness Index (S) and Shannon-Weiner Index (*H*′) with the procedure described by Fedor and Spellerberg. [43]. The Species Richness Index (S) was obtained by counting the number of endophytic fungal species in the corresponding plant tissues. A total of 130 isolates were participated in the antimicrobial activities assessment in this study.

### 4.8. Statistical Analyses

All results were expressed as the mean ± SEM. Graphs were prepared using GraphPad Prism 5.0. (GraphPad Software, San Diego, CA, USA) Significant differences between treatments were evaluated by the one-way analysis of variance (ANOVA) and the least significant difference (LSD) test at *p* < 0.05 using SPSS 19.0 (IBM, Armonk, NY, USA).

## 5. Conclusions

In this study, the diversity and antimicrobial activities of the endophytic fungi from *C. japonicus* were investigated for the first time. Our results illustrated that *C. japonicus* harbored abundant fungal endophytes representing a high level taxonomic diversity. It was found that 13 out of 130 endophytic fungal ethyl acetate extracts exhibited inhibitory activities against at least one of the pathogenic microorganisms. Specifically, the strain F8158, which was identified as *Trichoderma* cf. *harzianum* (commonly used as a biocontrol strain in the agricultural production process), exhibited good antagonistic activity against different plant pathogenic fungi and has a potential application in the biological control. In addition, it is noteworthy that the strain F8157 (*Thanatephorus cucumeris*, an opportunistic pathogen) showed antibacterial and antifungal activity firstly and has the potential research value. Overall, it was indicated that the endophytic fungi from *C. japonicus* may be of potential interest in screening bio-control agents and discovery of new bioactive compounds.

## Figures and Tables

**Table 1:** Identification of endophytic fungi from *C. japonicus* by the Basic Local Alignment Search Tool (BLAST) in the GenBank.

No.	Closest Species	Isolates Numbers	Identity (%)	N	IF
1	*Arthrinium hydei* CBS 114.990 (KF144890)	F8164	95.42	1	0.30
2	*Aspergillus flavipes* AfH14F02 (MK952227)	F8142, F8204, F8225	98.73	3	0.90
3	*Aspergillus terreus* MD32_7 (JQ697508)	F8121, F8159, F8172, F8191	99.46	20	6.02
4	*Aspergillus pseudoglaucus* ALE-85 (MF380824)	F8123	99.24	1	0.30
5	*Aspergillus niger* strain H1 (KJ778683)	F8143, F8150, F8186	99.30	6	1.81
6	*Aspergillus oryzae* isolate 118 (MH345958)	F8160, F8161, F8224	99.82	4	1.20
7	*Aspergillus* sp. isolate SW511 (MH509427)	F8190, F8243, F8238	99.47	3	0.90
8	*Aspergillus tubingensis* BSZ-6(2) (KJ190960)	F8208	99.81	1	0.30
9	*Aspergillus flavus* strain S2599 (MG575474)	F8111	99.64	1	0.30
10	*Clonostachys rosea* QLF2 (FJ025204)	F8195	99.26	1	0.30
11	*Thanatephorus cucumeris* AG-7 (AY154305)	F8154, F8157	98.71	6	1.80
12	*Podospora nannopodalis* CBS 113.680 (MH862937)	F8109	99.02	1	0.30
13	*Podospora setosa* CBS 613.84 (MH861787)	F8117, F8156	99.21	3	0.90
14	*Cladosporium tenuissimum* 7P6 (KY400093)	F8144	99.81	1	0.30
15	*Diaporthe eres* HUTA286 (KU377286)	F8107, F8205	99.26	7	2.11
16	*Diaporthe longicolla* ALE-196 (MF380785)	F8124	97.95	1	0.30
17	*Diaporthe nobilis* ZZDN1 (MG736062)	F8128, F8196, F8207	99.28	5	1.51
18	*Diaporthe gulyae* SJC3-2 (KX077244)	F8116, F8179, F8192	99.63	11	3.31
19	*Diaporthe longicolla* ALE-196 (MF380785)	F8124	97.95	1	0.30
20	*Diaporthe vaccinii* Lan1 (KC488258)	F8137, F8180, F8206	99.11	3	0.90
21	*Diaporthe citrichinensis* ZJUD034B (KJ210539)	F8193, F8194, F8237	97.32	3	0.90
22	*Stagonosporopsis cucurbitacearum* D49 (MF401570)	F8151	99.06	1	0.30
23	*Didymella bellidis* YS24 (MN443603)	F8165	100.00	1	0.30
24	*Dothiorella gregaria* hz-S8 (FJ517548)	F8178	100.00	1	0.30
25	*Paraconiothyrium brasiliense* 1-53 (JF502455)	F8181	99.62	1	0.30
26	*Colletotrichum boninense* JL7 (KM513575)	F8108	99.82	1	0.30
27	*Colletotrichum* sp. SL10 (KP689235)	F8110, F8135, F8129, F8170, F8176, F8201, F8210, F8213, F8235, F8115, F8120	99.10	31	9.34
28	*Colletotrichum acutatum* JS4 (KM513609)	F8114, F8118, F8129, F8134, F8140, F8152, F8162, F8167, F8188, F8190, F8198, F8231	100.00	55	16.57
29	*Colletotrichum truncatum* SL3-CJL17 (KP900265)	F8119, F8141, F8171, F8174, F8175, F8177, F8187, F8202, F8211, F8212, F8221, F8244	99.81	109	32.83
30	*Colletotrichum fructicola* JXNC-7 (MK041519)	F8136, F8155	99.81	2	0.60
31	*Colletotrichum gloeosporioides* HBxn-3 (HQ645077)	F8203	99.26	1	0.30
32	*Colletotrichum higginsianum* XN4-5 (JF830783)	F8239, F8240	99.63	2	0.60
33	*Trichoderma* cf. *harzianum* PDAN04-1 (MF108878)	F8158	99.66	2	0.60
34	*Trichoderma* sp. SDAS203699 (MK870422)	F8166, F8182, F8197, F8220, F8223, F8226	98.99	6	1.81
35	*Hypoxylon fragiforme* RY-3 (MK429859)	F8218	98.97	1	0.30
36	*Ceriporia alachuana* CEAL72 (MF358878)	F8112	99.36	1	0.30
37	*Mucor fragilis* BC3 (MK910073)	F8199	99.50	1	0.30
38	*Muyocopron lithocarpi* MFLUCC:17-1500 (MT137781)	F8248	99.82	1	0.30
39	*Septoria arundinacea* LHH10-8 (JX077003)	F8125, F8185, F8219, F8246	100.00	4	1.20
40	*Fusarium sambucinum* CBS 146.95 (KM231813)	F8126, F8132	98.79	2	0.60
41	*Fusarium verticillioides* G4 (MK264336)	F8200	100.00	1	0.30
42	*Calonectria eucalypti* (KM357290)	F8168	99.03	1	0.30
43	*Ilyonectria robusta* (MN121555)	F8145	99.40	1	0.30
44	*Dactylonectria alcacerensis* JZB3310013 (MN944923)	F8217	99.42	1	0.30
45	*Nectria pseudotrichia* (MN121548)	F8131	98.58	2	0.60
46	*Neonectria* sp. nc_gw_9967b (KF428559)	F8153	98.82	1	0.30
47	*Plectosphaerella* sp. GZUIFR-QL9.9.1 (MK880441)	F8247	98.86	1	0.30
48	*Alternaria alternata* Acf-4 (MK795217)	F8214, F8215	99.63	2	0.60
49	*Talaromyces funiculosus* S01324 (MG744693)	F8138, F8149	99.46	2	0.60
50	*Talaromyces pinophilus* KR9 (MF153381)	F8230	99.83	1	0.30
51	*Talaromyces purpureogenus* NFML_CH66 (KM458841)	F8222	98.73	1	0.30
52	*Nemania* sp. 1 XS-2016 VD026 (KT588467)	F8183, F8229, F8113	99.26	3	0.90
53	*Biscogniauxia* sp. strain LPS-70 (MF379340)	F8130, F8147	99.63	2	0.60
54	*Leptospora rubella* strain ZLVG 319 (HE774478)	F8127	96.38	1	0.30
55	*Ascomycota* sp. isolate FL15 (MK370697	F8146	90.00	1	0.30
56	Sordariomycetes sp. 329 isolate FL0587 (JQ760299)	F8184, F8209, F8233, F8241, F8245	86.76	5	1.51
Total	130	130		332	100
Species Richness (S)		56			
Shannon-Wiener index (*H*′)		2.7076			

**Table 2:** Taxa of all endophytic fungi isolates from *C. japonicus*.

No.	Phylum	Class	Order	Family	Genus	Number (N)
1	Ascomycota	Sordariomycetes	Xylariales	Apiosporaceae	Arthrinium	1
2			Hypocreales	Xylariaceae	Nemania	3
3					Biscogniauxia	4
4				Hypoxylaceae	Hypoxylon	1
5				Bionectriaceae	Clonostachys	1
6				Nectriaceae	Fusarium	3
7					Calonectria	1
8					Ilyonectria	1
9					Dactylonectria	1
10					Nectria	1
11					Neonectria	1
12				Hypocreaceae	Trichoderma	8
13			Sordariales	Chaetomiaceae	Podospora	4
14			Diaporthales	Diaporthaceae	Diaporthe	31
15			Glomerellales	Glomerellaceae	Colletotrichum	201
16				Plectosphaerellaceae	Plectosphaerella	1
17		Eurotiomycetes	Eurotiales	Aspergillaceae	Aspergillus	39
18				Trichocomaceae	Talaromyces	4
19		Dothideomycetes	Capnodiales	Cladosporiaceae	Cladosporium	1
20					Leptospora	1
21			Pleosporales	Mycosphaerellaceae	Septoria	4
22				Didymellaceae	Stagonosporopsis	1
23					Didymella	1
24					Dothiorella	1
25				Pleosporaceae	Alternaria	2
26				Didymosphaeriaceae	Paraconiothyrium	1
27			Muyocopronales	Muyocopronaceae	Muyocopron	1
28	Basidiomycota	Agaricomycetes	Cantharellales	Ceratobasidiaceae	Thanatephorus	6
29			Polyporales	Irpicaceae	Ceriporia	1
30	Mucoromycota	Mucoromycetes	Mucorales	Mucoraceae	Mucor	1
Total	3	5	12	21	30	325

**Table 3:** Antimicrobial activities of culturable endophytic fungi from *C. japonicus*.

Isolates No.	Taxa (Accession Number)	Inhibition Zone in Diameter on Petri Plate (mm)						
		*B. cereus*	*E. coli*	*B. subtilis*	*S. aureus*	*P. aeruginosa*	*R. solanacearum*	*C. albicans*
F8107	*Diaporthe eres* (MN429162)	12.02 ± 0.11	10.44 ± 0.04	10.93 ± 0.05	13.27 ± 0.16	-	10.10 ± 0.07	-
F8121	*Aspergillus terreus* (MN429176)	17.14 ± 0.13	10.08 ± 0.14	20.65 ± 0.40	16.12 ± 0.08	-	-	9.09 ± 0.04
F8127	*Leptospora rubella* (MN429181)	11.34 ± 0.12	-	14.26 ± 0.09	12.58 ± 0.15	-	8.96 ± 0.08	-
F8157	*Thanatephorus cucumeris* (MN429209)	-	-	12.14 ± 0.14	-	10.32 ± 0.20	8.27 ± 0.18	8.94 ± 0.05
F8158	*Trichoderma* cf. *harzianum* (MN429210)	19.15 ± 0.13	10.81 ± 0.28	19.12 ± 0.15	19.36 ± 0.14	10.21 ± 0.12	12.02 ± 0.03	10.81 ± 0.16
F8159	*Aspergillus terreus* (MN429211)	15.33 ± 0.13	10.76 ± 0.25	21.15 ± 0.27	17.37 ± 0.11	-	16.48 ± 0.13	9.75 ± 0.17
F8160	*Aspergillus oryzae* (MN429212)	11.21 ± 0.14	-	12.13 ± 0.10	12.12 ± 0.08	-	11.02 ± 0.03	-
F8172	*Aspergillus terreus* (MN429223)	17.31 ± 0.19	10.84 ± 0.03	20.82 ± 0.17	16.39 ± 0.09	-	12.46 ± 0.10	10.21 ± 0.12
F8189	*Colletotrichum acutatum* (MN429239)	12.30 ± 0.17	-	16.04 ± 0.13	13.17 ± 0.18	-	11.05 ± 0.09	9.14 ± 0.07
F8191	*Aspergillus terreus* (MN429241)	20.56 ± 0.30	-	20.62 ± 0.41	15.38 ± 0.08	-	11.28 ± 0.15	9.34 ± 0.12
F8204	*Aspergillus flavipes* (MN429254)	12.29 ± 0.18	-	10.27 ± 0.18	-	-	8.33 ± 0.13	-
F8219	*Septoria arundinacea* (MN429268)	10.21 ± 0.13	-	10.98 ± 0.08	11.29 ± 0.15	-	10.85 ± 0.12	
F8225	*Aspergillus flavipes* (MN429274)	11.13 ± 0.12	-	15.31 ± 0.24	16.23 ± 0.12	-	13.94 ± 0.08	-
Positive control-1	Ampicillin sodium	20.48 ± 0.53	15.46 ± 0.22	23.34 ± 0.38	21.49 ± 0.27	17.34 ± 0.26	19.55 ± 0.21	-
Positive control-2	Actidione	-	-	-	-	-	-	21.15 ± 0.84
Negative control	Methanol	-	-	-	-	-	-	-

## Supplementary Materials

The following are available online at https://www.mdpi.com/1422-0067/21/17/5958/s1, can be found at the supplementary files. Supplemental files 1. Thanatephorus cucumeris AG-7 (AY154305). Supplemental files 2. Maximum-likelihood phylogenic analyses by internal transcribed spacer (ITS) sequence alignment for the endophytic fungi from *Chloranthus japonicus* Sieb belonging to Apiosporaceae (A); Aspergillaceae (B); Bionectriaceae (C); Ceratobasidiaceae (D); Chaetomiaceae (E); Cladosporiaceae (F); Didymellaceae (G); Diaporthaceae (H); Glomerellaceaee (I); Hypocreaceae (J); Didymosphaeriaceae (K); Hypoxylaceae (L); Irpicaceae (M); Mucoraceae (N); Muyocopronaceae (O); Mycosphaerellaceae (P); Nectriaceae (Q); Plectosphaerellaceae (R); Pleosporaceae (S); Trichocomaceae(T); Xylariaceae (U). Supplemental files 3. The raw data for antimicrobial activity.

## Abbreviations

BLAST	Basic Local Alignment Search Tool
IF	Isolation frequency
ITS	Internal transcribed spacer
PCR	Polymerase chain reaction
RA	Relative abundance
SSA	Similarity threshold
